# Vaccination and Thrombotic Thrombocytopenic Purpura

**DOI:** 10.4274/tjh.galenos.2020.2020.0060

**Published:** 2020-08-28

**Authors:** İrfan Yavaşoğlu

**Affiliations:** 1 Adnan Menderes University Faculty of Medicine, Division of Hematology, Aydın, Turkey

**Keywords:** Vaccination, Thrombotic thrombocytopenic purpura

## To the Editor,

The article entitled “Diagnostic Testing for Differential Diagnosis in Thrombotic Microangiopathies,” written by Zini and De Cristofaro [1] and published in one of the recent issues of your journal, was quite interesting. Herein, I wish to contribute to that article.

In the adult age group, vaccines did not contribute to the development of immune thrombocytopenia (ITP), but an increase was reported in diphtheria-tetanus-pertussis-poliomyelitis vaccines without statistical significance [2]. Immune-origin thrombocytopenia may be developed after many vaccines such as measles-mumps-rubella and varicella, polio, rabies, and meningococcal C, especially in childhood. This occurs with 1-3/100,000 vaccine doses. Molecular mimicry theory is thought to play a role in the development of ITP [3]. In adult cases, development of thrombocytopenic thrombotic purpura (TTP) has been reported with some vaccines [4,5,6,7,8,9]. These cases are summarized in Table 1. It is generally seen with vaccinations against viral agents. A frequent occurrence with vaccines against influenza may be relative due to more intensive vaccination. It is especially important within 2 weeks after vaccination.

Consequently, attention should be paid to the development of TTP after vaccination.

## Figures and Tables

**Table 1:**
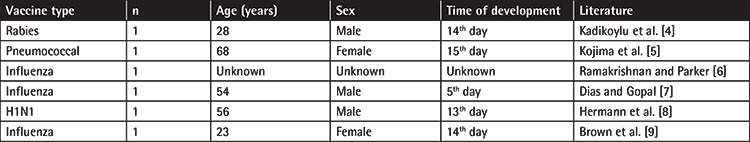
TTP cases developed after vaccination in the literature.
