# Supplementary material for: Vaccination and Thrombotic Thrombocytopenic Purpura
Source: Turk J Haematol. 2020 Aug 28;37(3):218–9. doi: 10.4274/tjh.galenos.2020.2020.0060 (PMC7463209; doi:10.4274/tjh.galenos.2020.2020.0060)
Supplement: Related texts

# Reply

## To the Editor,

Thank you for your communication.

The short letter that refers to our article provides some useful additional information and does not need any reply or comment.

**Best regards,**

Gina Zini and Raimondo De Cristofaro
